# The ‘costs’ of absorbent hygiene product mismanagement: A mini-review of contemporary literature from the Global South

**DOI:** 10.1177/0734242X261435293

**Published:** 2026-03-30

**Authors:** Marc Kalina, Sinethemba Dlamini, Catherina Schenck

**Affiliations:** 1 Centre for Interdisciplinary Studies of Children, Families, and Society, University of the Western Cape, Cape Town, South Africa; 2 Faculty of Law, Stellenbosch University, South Africa

**Keywords:** absorbent hygiene products, waste management, global South, environmental costs, environmental health, municipal solid waste

## Abstract

The mismanagement of absorbent hygiene product (AHP) waste – including disposable diapers, sanitary pads, and incontinence products – represents an emerging but understudied challenge across the Global South. Rapid urbanization and increased uptake of disposable hygiene products have outpaced the development of adequate waste management systems, leaving AHPs largely unsegregated, untreated, and unmanaged. This mini-review synthesizes contemporary literature from the Global South to explore the ‘costs’ associated with three common mismanagement scenarios: co-disposal with household waste, management outside formal systems, and non-management in the environment. Findings reveal that while the spaces of mismanagement are well documented, the costs remain poorly understood, unevenly distributed and often hidden. Co-disposal exacerbates landfill overcapacity, cross-contamination and greenhouse gas emissions; disposal outside waste systems burdens sanitation networks with blockages and infrastructure damage; and non-management disperses risks into public domains, contaminating soils and waterways, shortening pit latrine lifespans and compounding health hazards for households and sanitation workers. Across contexts, these costs are borne disproportionately by vulnerable groups, including women, children, animals, informal waste workers and low-income households, yet remain largely absent from policy debates and product life cycle assessments. Addressing these gaps requires more context-specific case studies, quantification of costs and greater recognition of disposal within sustainability frameworks to ensure equitable and effective interventions.

## Introduction

The mismanagement of absorbent hygiene product (AHP) waste – such as disposable diapers, sanitary pads and adult incontinence products – is a growing but largely overlooked environmental and public health challenge in the Global South. Rapid urbanization, population growth and increased use of disposable hygiene products, particularly in low- and middle-income countries, have outpaced the development of appropriate waste management infrastructure (Abhiharshan et al., 2025; Nagar, 2024; Shiam Babu et al., 2025; Thomas-Possee et al., 2024). As a result, AHP waste is frequently co-disposed with general household waste, dumped illegally or burned in open areas, contributing to land and water pollution, greenhouse gas emissions and human exposure to pathogens (Chitaka et al., 2025; Dutta et al., 2024; Sepadi, 2022; Turyahabwe et al., 2022). Municipal systems often lack the capacity, funding or policy frameworks to safely segregate, collect or treat this highly contaminated waste stream, while informal waste workers – who play a key role in recycling – typically avoid AHPs because of health risks and lack of market value (Niyobuhungiro et al., 2025; Purwati et al., 2021; Velasco Perez et al., 2021). As a result, the bulk of AHPs consumed within South countries remain unsegregated, untreated and unmanaged, posing significant health risks to waste workers, residents and the broader environment (Chitaka et al., 2025; Okotto-Okotto et al., 2024). Yet, although the problem is widely recognized, and remains a prominent point of discussion both in waste management literature as well as at international conferences such as the 2024 ISWA World Congress in Cape Town, South Africa, where AHP waste was prominently discussed, the true ‘costs’ of AHP waste mismanagement – spanning environmental degradation, public health impacts and burdens on municipal services – remain poorly quantified and inadequately understood. When AHP waste is mismanaged, as it broadly is across Global South contexts, what types of costs accrue, where and to whom?

The purpose of this mini-review was to explore understandings and identify knowledge gaps on the ‘costs’ of AHP waste mismanagement within the Global South.^1^ Specifically, we seek to understand the ‘costs’ associated with three scenarios of mismanagement. First, we aim to unpack the ‘costs’ associated with *the co-disposal of AHP waste alongside general household waste* – when AHP waste is collected and landfilled, rather than recycled, what impacts occur? Second, we want to untangle the ‘costs’ of *managing AHP waste outside waste management systems* – what are the costs to, dispose of, collect or clean-up AHP waste where formal waste systems do not exist, or once it has leaked into the environment? Third, we aim to unpack the ‘costs’ associated with the *non-management of AHP waste* – what are the short-term and long-term costs of leaving AHP waste in the environment over its lifespan? Within these three scenarios, we want to highlight directions of contemporary scholarship, as well as spaces for future investigation, on the types of costs accrued, where they occur, and to whom, with the goal of contributing to a more complete understanding of the true costs of AHP waste mismanagement within the Global South.

Our findings highlight that while AHP waste is increasingly recognized as a pressing challenge, it is rarely treated as a distinct waste stream in either scholarship or practice, and remains subsumed within broader discussions of household solid waste management. Yet, the results of this review show that mismanagement generates diverse and intersecting costs, borne by a multiplicity of actors – from households and informal workers to municipalities, sanitation providers and ecosystems themselves. Co-disposal exacerbates risks of landfill overcapacity, cross-contamination and greenhouse gas emissions; management outside waste systems burdens sewerage networks and sanitation providers with costly blockages and infrastructural failures; while non-management disperses risks into the public domain, polluting soils and waterways, shortening the lifespan of pit latrines and compounding health hazards for both communities, animals and sanitation labourers. This diversity of impacts underscores the distinctive burdens posed by AHP waste, while highlighting persistent challenges: inadequate empirical evidence, limited policy recognition and weak integration of disposal within product life cycle analyses. Although isolated studies – most notably from South Africa – offer national or local insight, comparative pictures remain rare, and the hidden costs borne by vulnerable actors remain largely unquantified. Key knowledge gaps concern the need to examine disposal impacts across varied contexts, to uncover and distribute hidden costs more equitably, and to position disposal more centrally in sustainability debates.

## Methodology

As noted, the purpose of this mini-review was to explore current understandings and identify knowledge gaps on the ‘costs’ of AHP waste mismanagement within the Global South. As such, our systematic search and selection process was designed to capture literature from within the Global South addressing the three specific mismanagement scenarios. Within these scenarios, the review sought to identify literature that spoke to the types of costs accrued, where they occur and to whom, as well as to highlight areas for further research.

### Defining ‘Cost’

Within our understanding, ‘cost’ refers to any sacrifice, trade-off or burden – material or immaterial – endured to achieve a goal, make a decision or carry out an action. Costs are shaped by perspective, time and distribution. What counts as a cost varies depending on who bears it – governments, businesses, communities or individuals – underscoring its subjective nature (Boardman et al., 2018). Actors linked to AHP waste have different goals and therefore accrue costs differently. For example, local governments mandated to provide waste services bear disposal and clean-up costs when AHPs are uncollected or dumped (Kalina et al., 2024). Individuals, meanwhile, seek safe and healthy communities, experiencing costs when disposing of waste themselves or encountering it in the environment (Kalina et al., 2022a, 2022b). Costs are also temporal, arising immediately (e.g., financial expenses) or over the long term, such as environmental degradation or chronic health conditions (Drummond et al., 2015). They are unevenly distributed, disproportionately affecting vulnerable groups like informal workers or low-income communities (Kalina, 2020, 2021; Kalina and Schenck, 2024). Many remain hidden from traditional assessments, including environmental externalities and unrecognized labour (OECD, 2018). A broader conception of cost, beyond finances, enables more equitable, sustainable and efficient decisions (Stiglitz et al., 2018), helping policymakers anticipate trade-offs and prioritize interventions that deliver overall value (OECD, 2018). Without this lens, crucial impacts may be overlooked, reinforcing inequalities and unsustainable choices.

### Search strategy

The review was conducted in accordance with the Preferred Reporting Items for Systematic Reviews and Meta-Analyses (PRISMA) 2020 statement. The review protocol was not formally registered. Eligible studies were required to address AHPs – including diapers, nappies, feminine hygiene products, wipes and other AHPs – in relation to costs, environmental impacts or other consequences connected to at least one of the three mismanagement scenarios described above. Only studies published in English between January 2020 and December 2025 were considered. Both peer-reviewed literature and high-quality grey literature, including theses and dissertations, were eligible, provided they met the quality criteria outlined below. Studies were excluded if they fell outside the date range, did not explicitly address AHP waste or were non-English.

The search was conducted exclusively in Google Scholar, which was selected for its broad multidisciplinary coverage, ease of access and ability to capture both peer-reviewed and relevant grey literature. Boolean search strings^2^ were developed using three thematic pools:

- **Theme 1** (conceptual/topic focus): *cost*, *impact*, *consequences*.
- **Theme 2** (product types): *diapers*, *diaper*, *nappy*, *nappies*, *feminine hygiene products*, *feminine hygiene product*, *wipes*, *absorbent hygiene products*, *absorbent hygiene product*.
- **Theme 3** (scenario terms): *mismanaged*, *mismanage*, *collect landfill*, *dump*, *dumped*, *dumpsite*, *clean-up*, *environment*, *lifespan*, *dispose*, *flush*.

Searches followed the format: (‘Theme 1’) AND (‘Theme 2’) AND (‘Theme 3’). In total, 351 unique term combinations were searched, covering the period from January 2020 to December 2025. Searches were run using Publish or Perish, a software programme that retrieves and analyses academic citations. Within each of the 351 unique Boolean combinations, only the first 1000 results were saved for screening.

The initial search retrieved 124,420 records. Following deduplication, 98,079 records were removed. Automated tools identified and excluded a further 561 records due to missing critical fields or falling outside the date range, and 227 records were excluded for being non-English. This left 25,433 records for screening. Screening took place in three stages. First, titles and abstracts were reviewed for relevance to the Boolean search themes and to the Global South context, retaining systematic reviews at this point; following this screening, 841 records remained. Second, full texts were assessed to determine whether AHP waste was explicitly discussed rather than mentioned in passing. Articles were retained if they included multiple references to AHP waste or dedicated sections on its role; systematic reviews were excluded at this stage if they did not specifically address the Global South. This resulted in 75 records. Third, all retained studies underwent quality assessment, with peer-reviewed articles included only if published in legitimate, indexed journals and grey literature scrutinized for methodological transparency and source credibility. As part of this assessment, articles from journals or publishers appearing in *Beall’s List of Potential Predatory Journals and Publishers* or *Cabell’s Predatory Reports* were excluded. The final dataset comprised 71 included studies.

Automated tools were used only during the identification stage to assist in removing duplicates and identifying ineligible records. All screening and quality assessment were conducted manually by the review team. Data from included studies were extracted into a structured spreadsheet capturing bibliographic information, study location, population or setting, waste types addressed, study design and key findings. Owing to heterogeneity in study designs and reported outcomes, a meta-analysis was not undertaken; instead, findings were synthesized narratively and thematically.

### Limitations

Only English-language literature was included in this review. This exclusion likely limits the scope and representativeness of the findings. As a result, important regional experiences, innovations or contextual nuances related to the costs of AHP waste mismanagement may not be reflected here. This language bias could skew the geographic distribution of evidence and underrepresent community-driven or locally adapted interventions. Future reviews should prioritize multilingual inclusion to improve comprehensiveness, capture a wider range of perspectives and enhance the applicability of findings across diverse global contexts.

## The literature

The 71 papers included in this review were read in full, with themes systematically coded in NVivo, a qualitative data analysis software developed by Lumivero.^3^ The captured literature spans a wide range of disciplines and methodological orientations, underscoring the breadth of scholarship on the topic. Although several coherent themes could be identified, broader trends regarding the scope and quality of the evidence were also evident. First, the majority of sources were peer-reviewed,^4^ while the remainder comprised theses, conference papers, preprints and technical or policy reports. Second, while the work spanned multiple disciplines, the primary studies were overwhelmingly qualitative, with only limited quantitative research represented. Next, although a large number of review articles were identified in the original screening, only a few (4) were included within the final sample, as most of those that were screened did not specifically address the Global South context or focus on AHP waste disposal practices and impacts. Finally, a geographical imbalance in the distribution of publications was also evident. As the review focused exclusively on the Global South, this concentration was expected; however, the literature was overwhelmingly clustered in Asian and African contexts. Only a small number of studies examined Global South populations situated within the Global North, for example, marginalized Indigenous communities in Australia or refugee camps in Greece (Hall et al., 2021; Manolakos, 2021).

Within the Global South, the distribution of studies was uneven. Research from Asia was concentrated largely in South and Southeast Asia, with countries such as India and Indonesia particularly prominent. In Africa, no contributions were identified from North Africa, and South Africa accounted for by far the largest share (12) of included publications. Other African countries were represented more sporadically, including Ghana, Nigeria and Kenya, but far less consistently. This imbalance may be indicative of a research gap, but may also be rooted in this review’s focus on English-language research, which may have excluded contributions from other research contexts. Furthermore, contributions tended to focus on contexts where AHPs were increasingly accessible, but where waste services were inadequate to manage growing volumes of AHP waste. For instance, studies in low-income or lower-middle countries like Ghana and Kenya often examined emerging, increasingly affluent urban populations beginning to adopt disposable products. In contrast, studies in middle-income or upper-middle income countries such as South Africa and India most often centred on underserviced urban populations, including slum dwellers, or unserviced rural communities. Rarely did the literature focus on adequately serviced communities, unless, as we will see, the specific emphasis was on the long-term impacts of the co-disposal of AHP waste with municipal solid waste.

The remainder of this review is organized thematically around three scenarios of AHP waste mismanagement. First, we consider the costs associated with co-disposal alongside general household waste, examining the impacts that arise when AHP waste is collected but landfilled rather than recycled. Second, we turn to the costs of management outside formal systems, assessing the burdens of disposal, collection or clean-up where waste services do not exist, or after AHP waste has leaked into the environment. Third, we explore the costs of non-management, interrogating the short- and long-term consequences of leaving AHP waste in the environment over its lifespan. Across these themes, the review seeks to show where the burden of improper disposal lies, what costs may arise, and who specifically bears those costs. The review concludes with a discussion that synthesizes the findings and highlights key research gaps, identifying critical directions for future studies.

## Co-disposal of AHP waste

The co-disposal of AHP waste with municipal solid waste emerged as the most common management pathway in contexts where households benefit from regular waste collection services. However, this practice is not regarded as best practice; separation at source and the development of dedicated treatment or recycling systems are increasingly recognized as preferable alternatives, as they mitigate environmental and public health risks while opening opportunities for resource recovery. Spaces of impact for the co-disposal of AHP waste with household waste typically include kerbside collection, within public bins, formal landfills as well as unsanitary dumpsites, and at home when AHP waste is burned or buried as part a household’s waste management strategy.

At kerbside, where refuse has been put out for collection, multiple actors were identified within the literature as being harmed by the co-disposal of AHP waste with household waste. First, studies consistently highlighted AHPs and faecal waste are frequently co-disposed with general refuse in unsanitary ways, with nappies, diapers and other faeces-containing materials potentially cross-contaminating the other waste fractions (Nayebare et al., 2020; Sprouse et al., 2022; Tilley and Kalina, 2020). For waste workers, but formal and informal, the potential health risks of encountering faecal matter or menstrual blood as well as from other cross-contaminated waste fractions were highlighted in multiple national contexts (Intauno and Poshai, 2023; Kisana and Shah, 2021; Senekane and Mngomezulu, 2024; Tatani, 2024). For instance, in South Africa, Senekane and Mngomezulu (2024) report that nearly half of informal waste pickers are exposed to biological risks during the course of their work including faeces, blood and dead animals. For sanitation workers in India, the improper disposal of diapers and pads in mixed waste streams underscores both the everyday physical risks and the entrenched social inequalities that deny workers the agency to challenge unsafe conditions (Kisana and Shah, 2021). Similarly, in Pakistan, Alam (2024) describes how rubbish bags are frequently torn open by children, animals or informal waste pickers before they can be collected, leaving pads exposed in public spaces, a challenge framed more as a matter of social or religious impropriety, which serves as source of stigma for menstruaters, than as an environmental concern.

Next, at landfill, costs have been found to be borne differently at sanitary landfills, engineered facilities designed with liners, leachate collection and controlled covering to safely isolate waste from the environment, and at unsanitary landfills which lack these protections, allowing uncontrolled leaching, odours and direct exposure to humans and ecosystems. Specifically, AHP waste represents a significant and growing burden on landfill capacity, with one review from South Africa noting they are the fourth largest contributor by volume to landfill space (Xulu et al., 2023). Disposable diapers are identified as one of the main fractions in municipal solid waste streams, with high per-capita use translating into millions of units annually confined to landfills in countries such as Indonesia (Elviliana et al., 2020). However, according to Blignaut et al. (2025) and Czarnecka et al. (2022) these characteristics have been found to underscore the environmental persistence of AHPs, which not only occupy scarce landfill airspace but also resist meaningful decomposition, exacerbating long-term management costs (Peter and Abhitha, 2021). This has become a growing concern as diaper waste emerges an increasing and sizeable share of municipal solid waste in South countries with one review estimating shares as large as 15% in México and 14.4% in Malaysia, highlighting the scale of the burden on local waste systems (Lim et al., 2022).

Beyond pressures on landfill capacity, the literature emphasizes the substantial carbon footprint of AHP disposal. Conventional sanitary pads, composed of up to 90% plastic, persist in landfills for centuries, compounding both airspace constraints and the sector’s carbon footprint (Panjwani et al., 2024). Landfilled diapers and pads also emit methane, compounding their global warming potential (Abd Manan et al., 2021; Huber, 2024). In Pakistan, where feminine hygiene products make up a significant share of municipal solid waste, poor disposal practices were estimated to release 22 Mt of CO_2_ in 2018 alone (Alam, 2024).

At unsanitary landfills, the literature consistently identifies leachate from unlined or poorly managed landfills as the principal concern, with far-reaching impacts on both soil and water systems. Unlike engineered sanitary sites, unsanitary landfills and open dump sites lack containment measures, allowing effluents laden with organic pollutants, pathogens, heavy metals and microplastics to infiltrate soils and aquifers (Nyamayedenga and Tsvere, 2020; Xulu et al., 2023). This uncontrolled leaching is strongly associated with freshwater and marine eutrophication, ecotoxicity and human carcinogenic toxicity, with unsanitary diaper disposal alone accounting for over 90% of measured aquatic ecotoxicity impacts in one life cycle assessment (LCA) from South Africa (Chitaka et al., 2025). Further research from Vietnam describes leachate contamination of groundwater with concentrations of heavy metals – including cadmium, lead, nickel and mercury – exceeding WHO drinking water standards by up to 10-fold (Giao et al., 2023a, 2023b). Alongside these water quality impacts, unsanitary landfills also contribute to greenhouse gas emissions, fires and explosions from uncontrolled gas build-up, compounding their environmental and public health risks (Xulu et al., 2023).

Finally, household-level burning and burying of AHPs is widely reported in contexts lacking formal waste management, but both practices carry significant environmental and health costs. Burial, while often chosen to remove waste from sight and reduce exposure to humans and animals, mirrors the risks of unsanitary landfilling: Leachate from decomposing diapers and pads readily infiltrates soils and groundwater, carrying pathogens and chemical residues that degrade water quality (Chitaka et al., 2025). Burning, though perceived as a convenient disposal method, is described both as inefficient – given the moisture content of excreta which complicates burning – and hazardous, releasing carcinogenic dioxins, greenhouse gases and toxic fumes linked to respiratory illness (Chitaka et al., 2025; Mlilo et al., 2021; Peter and Abhitha, 2021). Practical challenges, such as the high water content of diapers and other organic wastes, limit the effectiveness of household burning, often leading to indiscriminate dumping in rivers or public spaces when combustion proves unworkable (Nell et al., 2024; Saifulloh et al., 2021). Furthermore, the ash and residues that remain after incomplete combustion can further leach into water sources or be ingested by domestic animals, compounding public health risks (Chitaka et al., 2025). In India and elsewhere, the secret burning of sanitary pads is noted to exacerbate pollution, while in low-income contexts more broadly, the lack of infrastructure amplifies the hazards of open incineration and uncontrolled smoke emissions (Kaur, 2020; Tatani, 2024).

## Managing AHP waste outside of waste systems

Managing AHP waste outside formal waste management systems refers to the costs associated with disposing of, collecting or cleaning up AHP waste where no structured systems exist, or after it has leaked into the environment. In such cases, the responsibility for ‘management’ shifts to sanitation service providers, who must contend with blockages, pump failures and added maintenance costs, as well as the environmental and public health consequences of sewer overflows or untreated discharges.

The reviewed literature highlights that flushing AHPs into sanitation networks is a pervasive practice with far-reaching infrastructural and financial consequences. Alda-Vidal et al. (2020) estimate that around half of sewer blockages can be attributed to hygiene products disposed of via toilets, and although we do not have estimates for South countries, their calculation of costs of £88 million annually for the United Kingdom from sewer blockages suggests that costs may be comparable across countries with extensive sewerage networks. These costs are compounded by the need to repair damaged pumps and treatment equipment, with sanitation providers ultimately absorbing much of the financial and operational impact of these inappropriate disposal practices (Alda-Vidal et al., 2020). Sewerage systems in many contexts were engineered before the widespread availability of disposable hygiene products and were never intended to accommodate solids other than excreta (Alda-Vidal et al., 2020). Once flushed, products such as wipes, pads and nappies do not break down as advertised but instead absorb water, grease and other materials, forming blockages that are difficult to locate and resolve (Karadagli et al., 2021; Zhang et al., 2022). Infrastructural fragility is particularly acute in under-resourced or ageing systems, where small-bore pipes, inadequate maintenance regimes and limited monitoring capacity amplify the risks of blockages and overflows (Hall et al., 2021; Sreekumar and Pandey, 2024). Moreover, similar patterns are observed globally, where wipes, sanitary pads and related materials contribute to sewer blockages, basement backups and street overflows, placing significant burdens not only on wastewater authorities but also on affected households (Karadagli et al., 2021).

Although significant gaps remain, this literature hints to the serious costs that arise when AHP waste has to be mismanaged outside formal systems. When AHP waste is flushed, it not only causes blockages but also requires costly detection and removal, while damaging pumps and treatment plants (Hall et al., 2021).

## Non-management of AHP waste

The non-management of AHP waste refers to the short- and long-term costs that arise when products are left unmanaged in the environment. Flushed products – particularly wipes and sanitary pads – were also noted for their persistence in sanitation and aquatic environments. In addition, the disposal of AHP waste, most often feminine hygiene products, into pit latrines was widely reported, where such items accumulate and complicate subsequent management.

The reviewed literature consistently highlights the widespread practice of dumping AHP waste in unmanaged spaces. Although dumping has been widely observed across national contexts, it is most widely reported in South Africa where disposal has been documented in open fields, bushes, rivers, dry riverbeds and along roadsides (Acker-Cooper et al., 2023; Dladla et al., 2021; Schenck et al., 2023a, 2023b; Slekiene et al., 2024). In urban and peri-urban settings, diapers and sanitary pads are reported to be frequently found scattered in drains, stormwater channels and vacant lots (Schenck et al., 2023a; Slekiene et al., 2024), while in rural areas, dumping often occurs near homesteads, animal grazing areas and waterways (Kapanda, 2020; Kordecki et al., 2022). Evidence from India and Indonesia underscores that public spaces, including schoolyards and riverbanks, are also common repositories of discarded menstrual waste, while shoreline litter in Indonesia illustrates how riverine dumping flows into marine environments (Pakasi and Purnamasari, 2024; Williams et al., 2022).

The costs of such practices are significant and multifaceted. First, to the environment, the open dumping of AHP waste has been connected to the pollution of soil, water and air, with respondents in South Africa, Malawi and Indonesia linking AHP litter directly to ecosystem damage, loss of biodiversity and livestock exposure (Acker-Cooper et al., 2023; Aryanti et al., 2024; Kalina et al., 2022a; Kapanda, 2020). Diapers and pads discarded near or into rivers leach contaminants and pathogens, threatening drinking-water supplies (Chitaka et al., 2025; Huber, 2024; Roekruangri and Sanguanchue, 2023).

Social and economic burdens compound these environmental and health risks. Indiscriminate dumping undermines the image of communities, making them appear dirty and uninviting, while also placing stigma on residents forced to live among waste (Kalina et al., 2024; Schenck et al., 2023b). Children and animals were highlighted as particularly vulnerable, as discarded diapers may be ingested by dogs, and children were commonly observed to play near and amongst dumping grounds in Ghana, Malawi and South Africa (Appiah, 2020; Chitaka et al., 2025; Kalina et al., 2022a). For households reliant on contaminated rivers and soils, the risks extend to food and water security, with studies in Zimbabwe documenting diaper-linked microplastic pollution in agricultural land and associated losses in crop viability (Jerie et al., 2024).

A second space of non-management highlighted in the literature concerns the flushing of AHPs into toilets and sewer systems. As previously noted in this review, considerable costs are already associated with clearing blockages and repairing infrastructure caused by flushed items. As described, items such as wipes, sanitary pads and tampons are frequently disposed of in toilets, often because of limited disposal options, stigma or lack of privacy in sanitation facilities (Alda-Vidal et al., 2020; Manolakos, 2021; Sreekumar and Pandey, 2024). Unlike biodegradable toilet paper, most products are plastic-based or contain superabsorbent polymers, meaning they do not break down once flushed (Tsigkou et al., 2020). Instead, they accumulate in small-bore sewer systems, clogging pipes and reducing flow capacity (Sreekumar and Pandey, 2024). When these items bypass treatment plants, they are discharged into rivers, lakes and oceans, where they remain highly visible as sewage-related debris (Alda-Vidal et al., 2020).

More critically, flushed AHPs contribute to the growing problem of microplastics. Wipes, tampons and sanitary pads are manufactured from polyethylene, polyester and polypropylene, which fragment into microscopic fibres during wastewater treatment and are discharged with effluents (Lee et al., 2021). These microplastics are persistent: They accumulate in aquatic environments, re-enter terrestrial systems through sewage sludge used in agriculture and enter food chains through fish, crops and drinking water (Alda-Vidal et al., 2020).

A final space of non-management identified in the literature concerns the disposal of AHPs into pit latrines.^5^ Diapers, sanitary pads, tampons and other solid objects are frequently discarded into these facilities across a wide range of national contexts, particularly within sub-Saharan Africa (Budeli et al., 2020; Kalina et al., 2022b; Kapanda, 2020; Okotto-Okotto et al., 2024). In Uganda, for example, women reported disposing of menstrual materials in pit latrines both at home and in the workplace, often citing taboos around menstruation and fears of being seen as motivating this behaviour (Hennegan et al., 2020). Similarly, studies across sub-Saharan Africa note that menstrual waste is a common component of pit latrine contents, with significant implications for subsequent management (Purshouse, 2021; Tatani, 2024). First, the addition of non-biodegradable AHP waste accelerates the rate at which pits fill, shortening their functional lifespan and requiring more frequent, costly construction of replacement pits (Chitaka et al., 2025). The absorbent polymers in pads and diapers also solidify sludge, reducing its pumpability and frequently blocking emptying equipment (Tatani, 2024). In Kenya, for instance, plastics, glass, diapers and sanitary pads were cited as major reasons why manual emptying was favoured over mechanical methods in slum areas, as machines were unable to handle the volume of solid waste present (Owuor et al., 2023).

Second, pit latrine disposal of AHPs carries potential short- and long-term environmental risks. Pits themselves are prone to leaching, meaning diapers and pads disposed alongside faecal matter can contribute to groundwater contamination (Chitaka et al., 2025). In communities reliant on shallow wells or groundwater for domestic use, this risk is particularly acute. Moreover, menstrual waste within pits introduces additional biological hazards: Used pads saturated with blood and excreta harbour pathogens that increase the infection risks faced by sanitation workers during pit emptying and handling (Tatani, 2024). For instance, Tatani (2024) describe sanitation workers in Kisumu, Kenya reporting frequent encounters with menstrual waste in pits, describing increased risks of infections, environmental contamination and psychosocial stress linked to the stigma of handling such materials. In Uganda, women themselves noted the embarrassment and discomfort associated with pit disposal, yet continued the practice in the absence of safe alternatives (Hennegan et al., 2020). During the COVID-19 pandemic, people also disposed of masks and AHPs in pit latrines; while this practice further complicated pit management, in the context of heightened fear of infection it may have been perceived as the safest available option (Kalina et al., 2022b).

## Discussion and conclusions

Across the reviewed literature, the impacts of AHP waste mismanagement are recognized as broad – spanning environmental degradation, public health risks and financial burdens on households, communities and municipal systems – yet remain poorly understood in scope and magnitude. While harmful outcomes are consistently reported across co-disposal, management outside formal systems and non-management, evidence is fragmented and uneven, leaving major gaps in understanding how, where and to whom costs accrue.

A recurring challenge is that AHPs are rarely treated as a distinct waste stream. Despite their high moisture content, pathogen load, lack of recyclability and persistence, most national and municipal strategies subsume them under general household solid waste. This obscures their particular risks and limits targeted interventions. Similarly, although spaces of mismanagement are well identified – landfills, dumpsites, sewers, pit latrines and public environments – the associated costs are seldom quantified, with long-term impacts often hidden, externalized to marginalized groups or deferred to future generations.

Product LCAs reflect another gap: Disposal pathways in the Global South are frequently simplified or excluded, often assuming sanitary landfilling while overlooking open dumping, burning or pit latrine disposal that dominate in practice (Nagornyi, 2023; Somers et al., 2021). This misrepresentation both skews product sustainability profiles and erases community-level experiences of mismanagement. Knowledge imbalances compound the issue. South Africa provides rare national-level data, but most countries lack systematic evidence, constraining both cross-country comparisons and regionally appropriate policy responses.

To move forward, research must prioritize context-specific case studies that trace disposal impacts across diverse settings, from urban slums to rural households. Hidden costs borne by informal workers, sanitation labourers, women and low-income communities need to be uncovered and incorporated into assessments, while efforts to quantify financial, environmental and health-related costs are essential for robust policy development. Critically, disposal must be positioned more centrally within LCAs and sustainability frameworks to reflect the realities of Global South waste systems.

This review shows that while the spaces and practices of AHP mismanagement are well documented, the costs remain only partially understood. Addressing these gaps will not only advance scholarly understanding but also provide the evidence base for more equitable, sustainable and context-appropriate interventions that recognize and address the true burdens of AHP waste.

## Notes

1. We consider that the proper management of AHP waste would involve its systematic separation at source, diversion from general household waste streams and treatment through dedicated recycling or recovery pathways to minimize environmental and public health impacts.

2. A Boolean search string is a precise query formula combining keywords with operators primarily AND, OR and NOT to narrow or broaden search results in databases, search engines

3. NVivo is a qualitative data analysis software developed by Lumivero which enables researchers to organize, code, analyse and visualize unstructured data.

4. Fifty-seven journal articles and one book chapter, accounting for approximately 80% of the total

5. A pit latrine is a simple on-site sanitation facility consisting of a hole or pit in the ground covered by a slab or floor, used for the collection and containment of human excreta.
